# Supplementary material for: Physicochemical Profile of Antiandrogen Drug Bicalutamide: Solubility, Distribution, Permeability
Source: Pharmaceutics. 2022 Mar 18;14(3):674. doi: 10.3390/pharmaceutics14030674 (PMC8954523; doi:10.3390/pharmaceutics14030674)
Supplement: Supplementary file 1 [file pharmaceutics-14-00674-s001.zip › pharmaceutics-1629409-supplementary.pdf]

Supplementary Material

Physicochemical profile of antiandrogen drug bicalutamide: Solubility, distribution, permeability

Tatyana V. Volkova, Olga R. Simonova, Germen L. Perlovich*

G.A. Krestov Institute of Solution Chemistry RAS, 153045 Ivanovo, Russia.

*Corresponding author:1 Akademicheskaya str., 153045 Ivanovo, Russian Federation,

E-mail: glp@isc-ras.ru

Supplementary materials - content:

Figure S1. PXRD patterns on BCL calculated, raw and after the solubility experiments. S2
Figure S2. DSC curves for BCL raw and after the solubility experiments. S2
Figure S3. Absorption spectra of BCL in water, 1-octanol, n-hexane and ethanol. S3
Figure S4. BCL cumulative amount permeated. S3
Table S1. Density of the investigated solvents at different temperatures and pressure p = 0.1 MPa. S4
Table S2. The group contribution parameters and the associated molar volumes of BCL S4
Table S3. Experimental ($X_2^{\exp}$) and calculated (X_2^{cal}) mole fractions solubility of BCL in the selected solvents at different temperatures and pressure p = 100 kPa. S5
Table S4. The parameters of the modified Apelblat and van’t Hoff equations. S5
Table S5. The sublimation thermodynamic parameters taken from [12] S6
Table S6. Thermodynamic functions of BCL solubility and solvation processes in the solvents at the studied temperatures. S7-S8

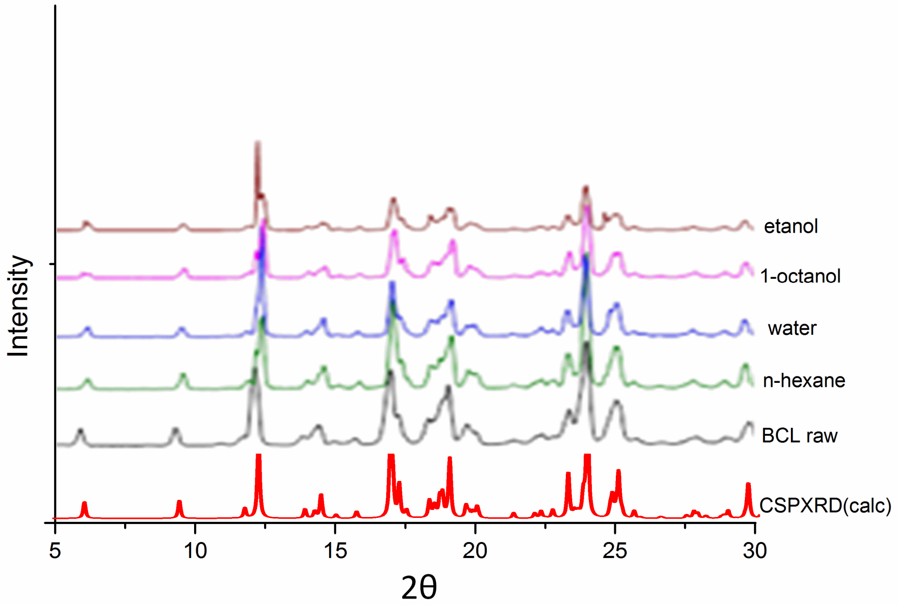

Figure S1. PXRD patterns on BCL calculated, raw and after the solubility experiments.

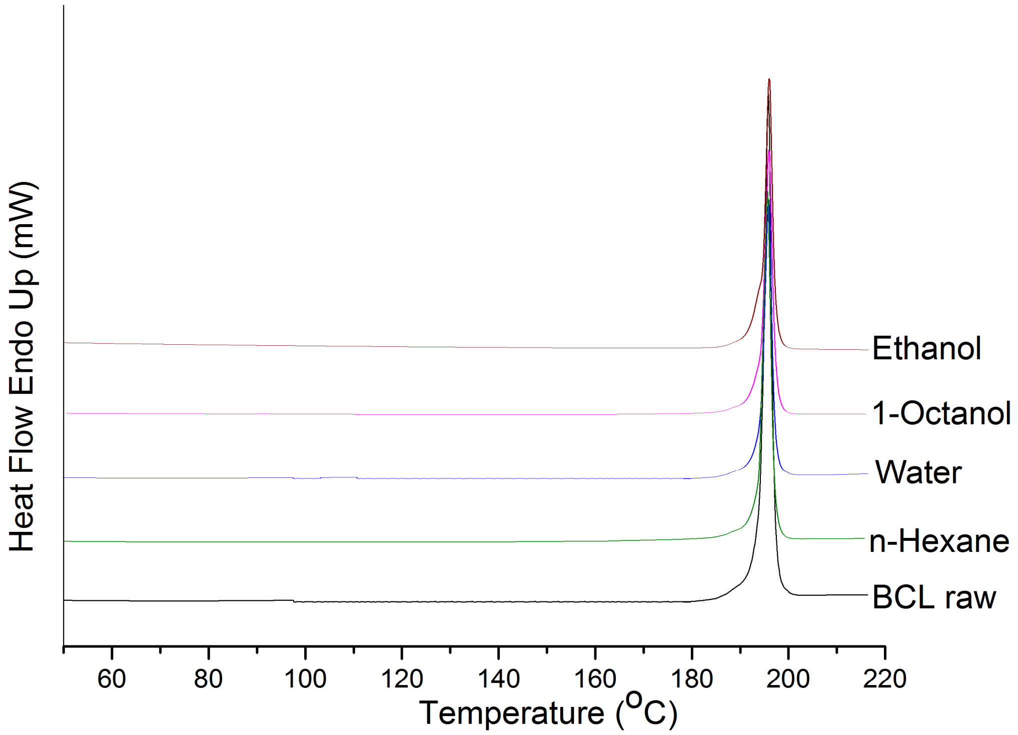

Figure S2. DSC curves for BCL raw and after the solubility experiments.

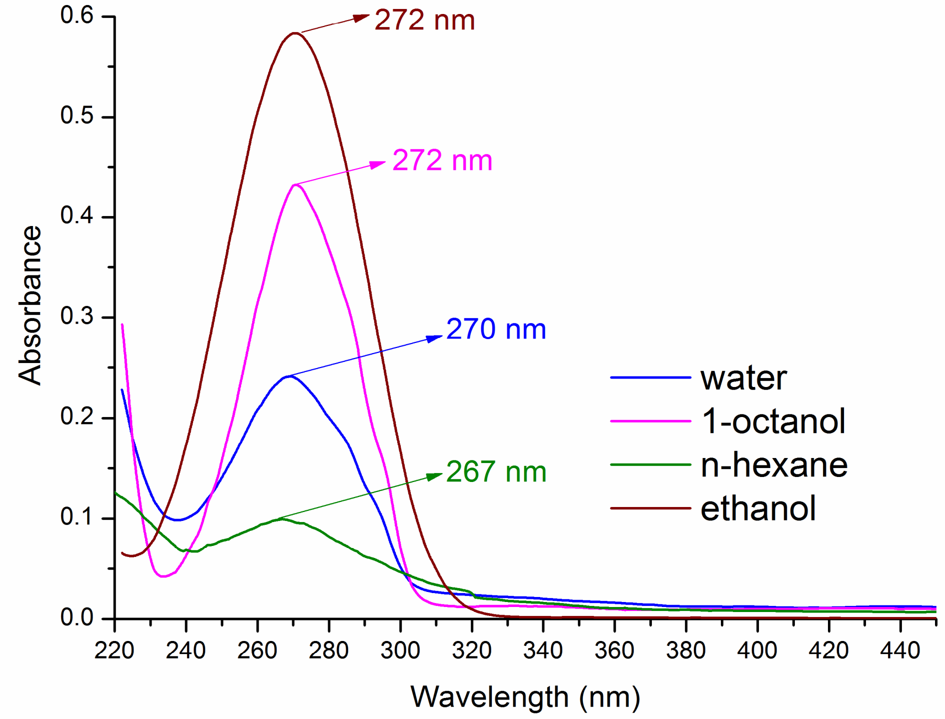

Figure S3. Absorption spectra of BCL in water, 1-octanol, n-hexane and ethanol.

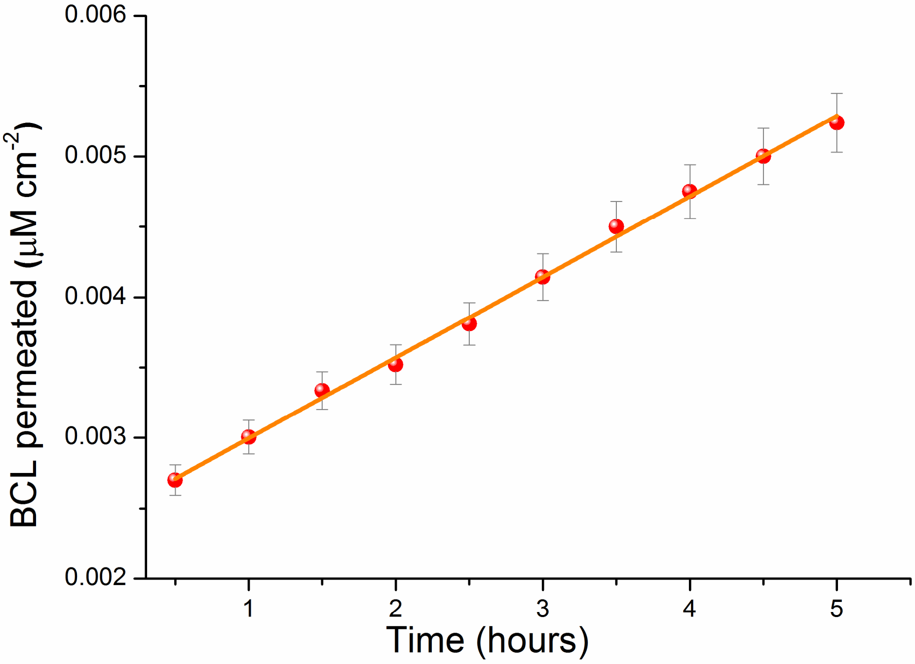

Figure S4. BCL cumulative amount permeated.

Table S1. Density of the investigated solvents at different temperatures and pressure p = 0.1 MPa.

Solvent	ρ/g·cm^{-3}					
	293.15 K	298.15 K	303.15 K	308.15 K	313.15 K	318.15 K
Water	0.9982	0.9968	0.9952	0.9936	0.9921	-
1-Octanol	0.8251	0.8217	0.8183	0.8148	0.8114	-
Hexane	0.6587	0.6543	0.6495	0.6453	0.6432	0.6361
Ethanol	0.7895	0.7852	0.7809	0.7766	0.7722	-

Table S2. The group contribution parameters and the associated molar volumes of BCL

Functional group	n	F_{di} ((J/m^3)$^{1/2}$·mol^{-1})	F_{pi} ((J/m^3)$^{1/2}$·mol^{-1})	E_{hi} (J/mol)	V_i (cm^3/mol)
			S-119		
-CN	1	430	1100	2500	24.0
$-CH_3$	1	420	0	0	33.5
$-CH_2-$	1	270	0	0	16.1
$-SO_2$	1	295.8	4361.0	200	51.0
>C<	2	-140(=-70×2)	0	0	13.0(=6.5×2)
-NH	1	160	210	3100	4.5
-OH	1	210	500	20000	10.0
-F	4	880(=220×4)	0	0	72.0(=18.0×4)
-CO	1	290	770	2000	10.8
phenylene	2	2540(=1270×2)	220=(110×2)	0	104.8(=52.4×2)
		5355.8	7161	27800	339.7

Table S3. Experimental ($X_2^{\exp}$) and calculated (X_2^{cal}) mole fractions solubility of BCL in the selected solvents at different temperatures and pressure p = 100 kPa.

T (K)	$X_2^{\exp}$	Modified Apelblat equation		van't Hoff equation	
		X_2^{cal}	$^a RD$ (%)	X_2^{cal}	$^a RD$ (%)
Water					
298.15	$1.13\cdot10^{-7}$	$1.12\cdot10^{-7}$	0.19	$1.15\cdot10^{-7}$	1.78
303.15	$1.47\cdot10^{-7}$	$1.44\cdot10^{-7}$	2.07	$1.48\cdot10^{-7}$	0.31
308.15	$1.90\cdot10^{-7}$	$1.84\cdot10^{-7}$	3.43	$1.88\cdot10^{-7}$	0.99
313.15	$2.35\cdot10^{-7}$	$2.33\cdot10^{-7}$	0.53	$2.39\cdot10^{-7}$	1.75
318.15	$2.97\cdot10^{-7}$	$2.95\cdot10^{-7}$	0.75	$3.00\cdot10^{-7}$	0.98
n-hexane					
293.15	$7.40\cdot10^{-7}$	$7.39\cdot10^{-7}$	0.20	$7.64\cdot10^{-7}$	3.19
298.15	$1.07\cdot10^{-6}$	$1.07\cdot10^{-6}$	0.20	$1.07\cdot10^{-6}$	0.36
303.15	$1.50\cdot10^{-6}$	$1.50\cdot10^{-6}$	0.32	$1.49\cdot10^{-6}$	0.97
308.15	$2.04\cdot10^{-6}$	$2.05\cdot10^{-6}$	0.27	$2.04\cdot10^{-6}$	0.15
313.15	$2.76\cdot10^{-6}$	$2.74\cdot10^{-6}$	0.65	$2.77\cdot10^{-6}$	0.52
318.15	$3.63\cdot10^{-6}$	$3.59\cdot10^{-6}$	1.23	$3.73\cdot10^{-6}$	2.85
1-octanol					
293.15	$2.23\cdot10^{-4}$	$2.22\cdot10^{-4}$	0.86	$2.25\cdot10^{-4}$	0.49
298.15	$2.57\cdot10^{-4}$	$2.57\cdot10^{-4}$	0.32	$2.61\cdot10^{-4}$	1.27
303.15	$2.99\cdot10^{-4}$	$2,96\cdot10^{-4}$	1.26	$3.01\cdot10^{-4}$	0.35
308.15	$3.40\cdot10^{-4}$	$3.41\cdot10^{-4}$	0.19	$3.46\cdot10^{-4}$	1.69
313.15	$3.95\cdot10^{-4}$	$3.91\cdot10^{-4}$	0.99	$3.96\cdot10^{-4}$	0.19
Ethanol					
293.15	$1.15\cdot10^{-3}$	$1.15\cdot10^{-3}$	0.59	$1.17\cdot10^{-3}$	1.95
298.15	$1.42\cdot10^{-3}$	$1.39\cdot10^{-3}$	2.12	$1.43\cdot10^{-3}$	0.28
303.15	$1.74\cdot10^{-3}$	$1.68\cdot10^{-3}$	3.59	$1.73\cdot10^{-3}$	0.81
308.15	$2.11\cdot10^{-3}$	$2.03\cdot10^{-3}$	3.82	$2.08\cdot10^{-3}$	1.22
313.15	$2.43\cdot10^{-3}$	$2.45\cdot10^{-3}$	0.75	$2.50\cdot10^{-3}$	2.72

$^a RD$ is the relative deviation: $RD = (X_2^{\exp} - X_2^{cal}) / X_2^{\exp} \cdot 100\%$;
Standard uncertainities: $u(T)$ = 0.15 K and $u(p)$ = 3 kPa;
Relative standard uncertainties for solubility: $u_r(X_2)$ = 0.045 for water and n-hexane; $u_r(X_2)$ = 0.04 for ethanol and 1-octanol.

Table S4. The parameters of the modified Apelblat and van't Hoff equations

Solvents	*A*	*B*	*C*	RMSD	RAD
Modified Apelblat equation					
Water	-80 ± 66	-864 ± 3013	11.8 ± 9.9	$3.4\cdot10^{-9}$	$1.4\cdot10^{-2}$
n-Hexane	361 ± 29	-22030 ± 1354	-52.9 ± 4.4	$2.7\cdot10^{-8}$	$1.9\cdot10^{-2}$
1-Octanol	-44 ± 25	-609 ± 1164	6.6 ± 3.8	$2.6\cdot10^{-6}$	$7.2\cdot10^{-3}$
Ethanol	-142 ± 79	3213 ± 3598	22.0 ± 11.8	$4.8\cdot10^{-5}$	$2.2\cdot10^{-2}$
van't Hoff equation					
Water	-0.9 ± 0.3	-4425 ± 77		$2.6\cdot10^{-9}$	$1.1\cdot10^{-2}$
n-Hexane	6.1 ± 0.3	-5919 ± 82		$5.3\cdot10^{-8}$	$5.7\cdot10^{-2}$
1-Octanol	0.5 ± 0.1	-2601 ± 43		$3.1\cdot10^{-6}$	$8.0\cdot10^{-3}$
Ethanol	5.1 ± 0.4	-3481 ± 114		$3.4\cdot10^{-5}$	$1.4\cdot10^{-2}$

Table S5. Melting and sublimation thermodynamic functions of Bicalutamide [12]

T_m^{onset} (K)	465.5 ± 0.2
ΔH_m (kJ·mol^{-1})	49.5 ± 0.5
ΔS_m (J·K^{-1}·mol^{-1})	106 ± 1
ΔG_{sub}^0 (kJ·mol^{-1})	63.7
ΔH_{sub}^T (kJ·mol^{-1})	117.1 ± 0.6
ΔH_{sub}^0 (kJ·mol^{-1})	124.7 ± 0.6
$T\Delta S_{sub}^0$ (kJ·mol^{-1})	61.0
ΔS_{sub}^0 (J·K^{-1}·mol^{-1})	204.6 ± 5.0

Table S6. Thermodynamic functions of BCL solubility and solvation processes in the solvents at the studied temperatures.

Temperature (K)	ΔG^0_{sol} (kJ·mol^{-1})	ΔH^0_{sol} (kJ·mol^{-1})	$T\Delta S^0_{sol}$ (kJ·mol^{-1})	ΔS^0_{sol} (J·mol^{-1}·K^{-1})	$-\Delta G^0_{solv}$ (kJ·mol^{-1})	$-\Delta H^0_{solv}$ (kJ·mol^{-1})	$-T\Delta S^0_{solv}$ (kJ·mol^{-1})	$-\Delta S^0_{solv}$ (J·mol^{-1}·K^{-1})	$^a\zeta_{H\,solv}$ (%)	$^b\zeta_{TS\,solv}$ (%)
					Water					
293.15	39.00		-2.20	-7.50±0.3	24.70		63.20	215.59	58.17	41.83
298.15	39.00		-2.20	-7.37±0.3	24.70		63.20	211.97	58.17	41.83
303.15	39.00	36.8±0.6	-2.20	-7.27±0.3	24.70	87.9	63.20	208.48	58.17	41.83
308.15	39.11		-2.31	-7.49±0.3	24.59		63.31	205.45	58.13	41.87
313.15	39.13		-2.33	-7.43±0.3	24.57		63.33	202.24	58.12	41.88
					1-Octanol					
293.15	20.49		1.11	3.79±3.6	43.21		59.89	204.30	63.26	36.74
298.15	20.49		1.11	3.73±3.6	43.21		59.89	200.87	63.26	36.74
303.15	20.45	21.6±0.4	1.15	3.81±3.6	43.25	103.1	59.85	197.43	63.27	36.73
308.15	20.46		1.14	3.70±3.6	43.24		59.86	194.26	63.27	36.73
313.15	20.40		1.20	3.82±3.6	43.30		59.80	190.96	63.29	36.71
					n-Hexane					
293.15	34.40		14.80	50.47±3.4	29.30		46.20	157.60	62.04	37.96
298.15	34.20		15.02	50.70±3.4	29.52		45.88	153.88	62.20	37.80
303.15	33.80		15.40	50.81±3.4	29.90		45.60	150.42	62.35	37.65
308.15	33.56	49.2±0.7	15.64	50.75±3.4	30.14	75.5	45.36	147.20	62.47	37.53
313.15	33.33		15.87	50.69±3.4	30.37		45.13	144.2	62.59	37.41
318.15	33.13		16.07	50.50±3.4	30.57		44.93	141.22	62.69	37.31
					Ethanol					
293.15	16.50		12.40	42.29±5.1	47.20		48.60	165.79	66.34	33.66
298.15	16.20		12.65	42.43±5.1	47.45		48.34	162.17	66.46	33.54
303.15	16.01	28.9±0.9	12.89	42.52±5.1	47.69	95.8	48.11	158.70	66.57	33.43
308.15	15.78		13.12	42.56±5.1	47.92		47.88	155.38	66.68	33.32
313.15	15.67		13.23	42.24±5.1	48.03		47.77	152.55	66.73	33.27

$^a\zeta_{H\,solv} = (|\Delta H^0_{solv}|/(|\Delta H^0_{solv}|+|T\Delta S^0_{solv}|))\cdot 100\%$; $^b\zeta_{TS\,solv} = (|T\Delta S^0_{solv}|/(|\Delta H^0_{solv}|+|T\Delta S^0_{solv}|))\cdot 100\%$;
